# Views of Some British Architects

**Published:** 1913-02-22

**Authors:** 


### MODERN HOSPITAL CONSTRUCTION.

### Views of Some British Architects.

At the meeting of the Royal Institute of British Archi tects on Monday, the 17th inst., papers on this subject were read by Mr. A. Saxon Snell and Mr. William Milburn, jun., B.Sc.

### MR. SAXON SNELL, in his contribution, remarked that the promoters of the newest hospital were anxious that it should embody the latest improvements, and be just a little better than the last one built. Modern hospitals certainly showed evidence of thought and labour on the part of medical men and architects. In this matter the opinions of Miss Nightingale, De Chaumont, Galton, Tollett, Parkes, and others might be studied with advantage. We in this country had concentrated upon sanitary fittings and local cleanliness, and we took great care in excluding soil-tainted air from wards. On the Continent, better provision than here was made for space in and around ward blocks. We had to learn from France and Germany in this respect, and they from us in regard to sanitary fittings. The pavilion type of hospital dated from the Crimean War, and held the field to this day. Before that time hospitals were mere collections of rooms, with little or no arrangement specially adapted for sick people, and the administrative offices and sanitary conveniences were all in comunication with the sick rooms. During the last thirty years the advances in medicine and surgery, and especially in bacteriology, had had their effect upon the arrangement and construction of hospitals. Bacteriology had not discounted the value of fresh air and sunlight, but had rather explained and emphasised it. He had discarded sash-framed windows in favour of solid hard-wood or steel casements. Engineering now entered so much into the work and maintenance of hospitals that their buildings and equipment required much more room; moreover, the baths, physical exercise halls, laboratories, and research rooms each needed their quota of space. Two notable attempts within the last thirty years had been made in planning ward blocks, one to substitute circular wards for pavilions and the other to group all the wards together. There was also the design of glass-cased cubicles, as at the Pasteur Hospital in Paris, for treating different infectious diseases under one roof. The design and arrangement of modern hospitals had been dominated by the advance in the knowledge and practice of medicine and the paramount need for light, especially sunlight. Any building was an obstruction to light and air, but there must be some means of shelter against wind, rain, and extremes of temperature. Subject to these limitations, the more air and sunlight could be got into the wards the better. The great need was large areas of land and wide spacing of ward blocks. This country was somewhat niggardly in regard to the area of hospital sites, because land was costly, especially in and around cities, where the need was particularly great. In the planning of the Manchester Royal Infirmary, and of the not quite complete King's College Hospital, there was a desire for more land than could be had. Many years ago the minimum fixed as desirable was one acre to fifty patients, but in many places abroad there was a more generous basis. Instances of this were Nuremberg, 40; Berlin, 32; Charlottenburg, 37; St. Denis, 26; Montpelier, 27; and the Johns Hopkins Hospital, Baltimore, which had only 26 to the acre. In and around cities, hospitals might be placed with advantage in the centre of some of the public parks; but that would mean the overcoming of mountains of prejudice, and the charge that the parks were being made the hotbeds of disease. He quoted Miss Nightingale's experience in the Crimean War, that in the hospital tents, where the sick were almost without shelter, blankets, and proper food or medicines, the mortality was not more than one-half what it was at Scutari, but these tents had only a few beds in each. Mr. Snell gave other instances to the same purport. The design of a hospital should be the joint work of the architect and the physician or surgeon, each of whom had his own sphere, and would do well to keep within it. He acknowledged with gratitude the assistance he received in the building of Charing Cross Hospital from the late Dr. Murray and from Mr. Stanley Boyd, the senior surgeon, who was now present. He considered that the extreme height of 25 feet for a ward was unnecessary, especially as in enclosed spaces there was but little movement of air beyond a height of 12 feet from the floor level. He concluded by exhibiting and explaining a model plan of a ward unit. It comprised a main sick ward with twenty beds, and separation wards for four beds. A strong point was avoiding shadowing the ward windows from the direct rays of the sun; the angle of incidence of the sun's rays in the early morning and afternoon allowed penetration to a greater distance than when it was at the meridian. A large south window opening direct into the ward was provided above the roof of the day room. The windows of the main block were alternately casements or French windows carried down to the floor level, with a hopper light above, and sash windows. For heating, in addition to the central open fires, small radiators might be placed immediately under the sash windows.

MR. WILLIAM MILBURN'S

paper was illustrated by a splendid series of slides depicting hospitals in most civilised countries which the author had visited. His object was to discuss the general disposition of buildings in relation to one another, and the arrangement of the wards and their annexes, rather than to enter into details of construction and equipment. He reminded his hearers that hospitals, in addition to being primarily for the treatment of the sick, were centres for medical education, clinical study, research, and investigation. Each special department—ophthalmic, nose, throat and ear, children, gynæcological—was subdivided into a number of ward units, the accommodation of each unit usually comprising one large ward, a number of small ones, and the necessary service, sanitary and medical rooms. Attached to each department were the special rooms requisite for the particular disease, such as the operating theatres and the hydro and electro-therapeutic rooms. The separate departments were self-contained, and yet mutually dependent upon one another, and so organised and managed as to form a complete and organic whole. The author proceeded to show how Lister's discoveries, and the general development of the science of medicine, had determined the schemes of construction, and supported Mr. Saxon Snell's emphasis on the need for plenty of space and free interchange of pure air. In English hospitals the general arrangement of the rooms in the ward unit was the same. The large ward usually contained twenty-four beds, and at its southern extremity two disconnected sanitary towers were placed containing the baths, lavatories, and sanitary annexes, while between these towers was placed a balcony for open-air treatment. The recently constructed King's College Hospital would doubtless rank as one of the finest and most complete in the world. The primary function of the isolation hospital for infectious diseases was to prevent the spread of disease amongst the public at large, and the buildings for this purpose ranked among the finest of the medical institutions. At the present time interesting developments were taking place in the design of these institutions, based on the modern medical opinion that infection in a hospital was usually conveyed by contact, not by aërial convection. He quoted the Seacroft Hospital, Leeds, the City Hospital at Edinburgh, and the City Hospital at Liverpool. He reviewed the main characteristics of the hospitals in France, Belgium, Holland, Germany—he had not visited Austria—and then proceeded to discuss the general features of the modern American hospitals. In this comparison he had great praise for the German hospitals, such as the Virchow, which occupied a site of sixty-three acres, with accommodation for 2,000 patients. In regard to America, his impression, derived from an inspection of many of the hospitals erected during the last fifteen years, was that whilst, as buildings, most of them were excellent, a large number lacked many of the essential requirements and details necessary to a hospital from the medical and hygienic points of view. But land was exceedingly expensive. Still, the youth and wealth of the country, the immense population, the rapidly-growing cities, and the demands of medical education needed the erection of new hospitals, and the greatest attention was being devoted by the authorities to the subject of hospital design and construction. The Johns Hopkins Hospital, Baltimore, was still one of the best hospitals in the United States, and was one of the most celebrated pavilion hospitals in the world. Similar praise would be due to the hospital at Cincinnati, now approaching completion, which was built on a site of twenty-seven acres, and designed for 1,400 beds. In New York the land problem was a very real one, and a hospital was now in course of construction on a site 800 feet by 200 feet, and about four acres in extent, intended for 600 beds. But it had the advantage of adjoining the Hudson River and of fronting a small park. There was a general feeling in Germany that the total accommodation of one institution should not exceed 1,500 beds. He concluded by expressing the hope and feeling that in future the general hospitals designed to serve the great cities would be constructed in suburban districts and on generous sites.

MR. E. T. HALL, in proposing a vote of thanks to the readers of the papers, discussed the main considerations which actuated him in designing hospitals. He pointed out that many of one's wishes had to be greatly modified owing to the exigencies of land space, and indicated how, in his opinion, the disadvantages might be minimised. The contiguity of wide roads must be taken into account as a set-off to the disadvantages of confined space, and he instanced the new wing of the Homœopathic Hospital in London.

DR. BOOBBYER (Nottingham)

narrated his efforts as medical officer of health in the provision of accommodation for cases of infectious disease, and laid very great stress upon the free interchange of air in the treatment of cases, even those suffering from fevers with high temperatures. He emphasised the importance of the heat-regulating centre in the brain, and alluded to the statement by the late Captain Scott, in reference to a previous Polar expedition, that after enduring temperatures many degrees below zero, when the men came into regions where the temperature was about freezing-point, they basked outside their sleeping suits in a condition of great comfort. Coddling by the fire and alternating that with exposure was very harmful, and apparently delicate patients not only came to no harm when treated absolutely in the open air, but showed rapid improvement. And Dr. Parsons, sent from the Local Government Board to report upon the system, after spending two days at the institution and inspecting the various charts and hospital sheets, reported that he could trace no evidence of ill effects.

The discussion was concluded by Mr. Stanley Boyd, senior surgeon of Charing Cross Hospital, who confirmed the great importance of the utmost available light for operating, and expressed his views as to the best methods of heating and lighting the operating theatre.

